# Development of Thermal Resistant FDM Printed Blends. The Preparation of GPET/PC Blends and Evaluation of Material Performance

**DOI:** 10.3390/ma13092057

**Published:** 2020-04-29

**Authors:** Jacek Andrzejewski, Lidia Marciniak-Podsadna

**Affiliations:** 1 Institute of Materials Technology, Polymer Processing Division, Faculty of Mechanical Engineering, Poznan University of Technology, Piotrowo 3 Street, 61-138 Poznan, Poland; 2 Institute of Mechanical Technology, Faculty of Mechanical Engineering, Poznan University of Technology, Piotrowo 3 Street, 61-138 Poznan, Poland; lidia.marciniak-podsadna@put.poznan.pl

**Keywords:** additive manufacturing, thermoplastic polyesters, polycarbonate, mechanical properties, dimensional accuracy

## Abstract

The paper discusses the preparation of polymer blends based on the polyethylene terephthalate copolymer/polycarbonate (GPET/PC). Materials have been prepared in order to assess their applicability in the fused deposition modeling (FDM) 3D printing process. The tested key feature was the thermomechanical resistance, measured by head deflection temperature (HDT) and Vicat softening temperature (VST), the mechanical tests and dynamic mechanical thermal analysis (DMTA) were also performed. A clear relationship between the increasing content of PC in the blend properties was observed. DMTA analysis revealed significant changes in the glass transition temperature, which indicates the miscibility of this type of polymer system. The mechanical tests indicate a clear trend of stiffness and strength improvement along with the increasing share of PC phase in the structure. The increase in impact strength is also clear, however, compared to the results for a pure PC, the results obtained for GPET/PC blends are significantly lower. As part of the research, reference samples based on polyethylene terephthalate homopolymer (PET) and composite samples with addition of 10% talc were also prepared. The structure analysis for PET/PC(50/50) samples did not show miscibility. However, due to the formation of the PET crystalline phase, the thermomechanical resistance of these materials was visibly higher. Scanning electron microscopy (SEM) analysis confirmed a high degree of compatibility of the GPET/PC blend structure as indicated by the lack of visible signs of phase separation. This phenomenon is not observed for PET/PC blends, which confirms the different thermomechanical interactions of both tested polymer systems.

## 1. Introduction

The use of polymer blends is one of the simplest methods for polymer modification. Polymer blending process has been used since the beginning of the polymeric material application. The main advantage of this method is simple, economical methodology, usually conducted during a single cycle process using twin screw extruders.

The polycarbonate (PC)-based materials are one of the most popular type of polymer blends, applied in several areas of industry, such as automotive, electronics and machine building. Commercially available PC-based blends can be divided into two main categories. The first group refers to the blends with an addition of styrene polymers, like acrylonitrile-butadiene-styrene (ABS) [1,2,3,4] or acrylonitrile-styrene-acrylate ASA [5,6]. The second category consists of PC and different types of thermoplastic polyesters, out of which polyethylene terephthalate homopolymer (PET) [7,8,9,10] and polybutylene terephthalate PBT [11,12,13] are the most popular. However, there are several other types of polyesters, which have already been used for blending with PC. Some of them, like polytrimethylene terephthalate PTT [14,15,16] or polylactic acid PLA [17,18,19] can be considered as more sustainable, as they are partly or fully biobased materials. The use of a PLA resin as the investigated blend component was the initial intention during the planning of the present study. A recent publication confirmed the formation of super-toughened structures of PC/PLA blends [19,20,21], which is possible thanks to the addition of elastomeric impact modifiers and reactive extrusion process conducted at high temperatures (>250 °C). Unlike in our study, the dedicated manufacturing technique for PC/PLA blends was injection molding. Also, the PC content in most of the reported blends exceeds 60 wt.%, while for the lower PC content the blend properties were poor.

For many studies the main research goal is the reduction of polycarbonate viscosity, this goal is most often achieved by using a possible low content of the modifier to avoid deterioration of the polycarbonate properties [22,23,24]. The viscosity of materials intended for fused deposition modeling (FDM) printing is also one of the important parameters that can constitute a field for modification. Due to high viscosity of PC the plasticizing temperature can reach even 300 °C or more. However, the main issue regarding the printing of PC-based materials is the warping tendency. The PC glass transition temperature (Tg ≈ 150 °C) is the reason for the negative phenomena during the printing process; the effects are shrinkage and deformation of models, therefore the use of a heated chamber during the process is necessary. Apart from the difficulties related to PC printing, its high thermomechanical resistance has great advantages in the context of applications. An important context of research is the possibility of using developed blends in the biomedical industry. The current trend in implantology is the use of reverse engineering methods [25,26,27,28], which is done by designing implants based on the actual shape of the tissue defect. Due to the strength requirements, most bone implants are made using the metal powders techniques [29,30,31], while the FDM printing has a large share in the production of orthoses and stabilizers.

The use of polyethylene terephthalate copolymer (GPET) copolymer as the construction material has not been widely practiced in the industry, mainly due to the properties of this polyester, that is, low thermomechanical resistance and impact strength. The main application of the GPET copolymer is the production of films, transparent plates and thick-walled containers manufactured by the injection technique. The key parameter for these applications is the high transparency of GPET, which results from its amorphous structure. Unlike the PET homopolymer, the addition of glycol during the polymerization process causes the disappearance of the crystallization ability, therefore the structure of GPET products, even with a very thick wall of the product, remains fully amorphous.

Until now, studies into to the use of GPET-based blends can be considered as basic research, without focusing on applications in specific industrial technologies. One example is the study by Lam et al. [32], where GPET resin was blended with polyoxymethylene (POM). Most of the mechanical properties obtained for blends turned out to be worse those reported for pure polymers. Similar conclusions emerge from a study by Chen et al. [33], where GPET was blended with ABS terpolymer. An interesting example of research into the use of GPET is an article by Cicala et al. [34], who investigated the use of GPET as an additive to polyetherimide (PEI). It should be emphasized that polyethyleneimine PEI is a popular material in the FDM technique, intended mainly for advanced structural applications. In this case, however, the samples were prepared by the injection molding technique. The results indicate that the properties for PEI/GPET blends do not differ significantly from those obtained for commercial materials, in this case pure PEI (Ultem 9085). The topic of GPET/PET-based blends also includes recycling-related issues. The main reason is the widespread use of GPET in film coextrusion technology, where it is used as an external layer for sandwich film due to its excellent weldability [35,36,37,38,39]. Most of the available literature indicates numerous technological problems and the need to use compatibilizers for the GPET/PET blends. Only a few of these studies deal with GPET/PC type of polymer blends. The work conducted by Chen et. al. [40] deals with the structure orientation issue during the extrusion process, while the paper presented by Zhang et. al. [41] is covering the subject of a rubber toughening phenomenon for GPET/PC/elastomer system. The lack of literature reports suggests that the research topics presented in this work can be considered as novel.

Due to its processing properties, mainly low shrinkage, GPET has become one of the most popular materials used in 3D printing by the FDM method. In comparison with PLA, the most popular material used. GPET has only a slightly higher glass transition temperature (≈65 °C) and a higher range of plasticizing temperature (≈240 °C), while for most of PLA varieties, the glass transition and plasticity temperatures are 55 °C and 190 °C, respectively. All these features make a perfect fit to the requirements of the FDM technique and can be the starting point for attempts to use this copolymer as a base material for making compositions with technical polymers, whose processing properties make it difficult to use in 3D printing.

The main purpose of this study was to assess the applicability of the developed GPET/PC blend system in manufacturing products using the FDM technique. The main assumption for the project was the possibility of making models on standard FDM machines, equipped only with a heated table. Apart from the mechanical properties, the main evaluation criterion was thermomechanical resistance (head deflection temperature (HDT) and Vicat softening temperature (VST) measurements). Qualitative assessment of the prepared models was based on geometrical measurements carried out using an optical scanner. The thermomechanical characterization was supplemented with the dynamic mechanical thermal analysis DMTA, while the structure observations were performed with the use of scanning electron microscopy.

## 2. Materials and Methods

### 2.1. Materials

To prepare the blend, we used Macrolon 2205 PC type (BASF, Ludwigshafen, Germany) supplied by Albis Polska. PET-G resin was Estar 6763 (Eastman Chemicals, Kingsport, TN, USA). RamaPET N1 (Indorama Ventures, Wloclawek, Poland) bottle grade polyethylene terephthalate (PET) was also used during the study. GPET and PET were supplied by Hanex GTX. All types of polymers were supplied in the form of pellets. The mineral filler used for the composite blend preparation was pure talc supplied by Biomus (Lublin, Poland).

### 2.2. Sample Preparation

The blends were prepared with the use of a ZAMAK EH16D co-rotating twin screw extruder (Zamak-Mercator, Skawina, Poland). For all blends the operating screw speed was 100 rpm. Temperature profile, from the hopper to the die was as follows: 200–210–220–230–240–250–260–260–240 (die). The extruder stand was cooled in a water bath and pelletized. The FDM filament was prepared using single screw extruder Metalchem W25-30D (IMPiB, Torun, Poland). The dried pellets (24 h, 70 °C) were extruded using a 3 mm die nozzle. The extrusion temperature was 260 °C, while the screw speed was 20 rpm. The extruded strand was picked up by a conveyor belt and cooled by a set of fans. During this process, the filament was simultaneously stretched to achieve the final diameter of 1.75 mm. The final product was collected onto a spool using a winder. Since the pure PC 3D printed samples could not be prepared by the FDM method, the reference samples were prepared by injection molding. We used ES 80/20 HLS machine (Engel Austria GmbH, Schwertberg, Austria), while the following processing parameters—injection temperature = 280 °C; mold temperature = 60 °C; injection pressure = 1150 bar; holding pressure = 750 bar; holding/cooling time = 10/30 s.

The 3D printed samples were prepared with the use of a Prusa i3 MK3 machine (PrusaResearch, Prague, Czech Republic). The nozzle toolpath (g-code) was generated using the Slic3r PE software (1.41.2 version). The FDM process was conducted with the use of a 0.4 mm brass nozzle. The necessary specimens were prepared with 100% infill density, for each material a minimum of 20 dumbbell samples and 80 mm bars were prepared. Individual layers of filling were printed alternately at an angle of ±45 degrees to the longitudinal axis of the sample. The layer height was set to 0.15 mm, while the width was 0.4 mm. The model shell consisted of 2 layers of material (≈0.8 mm). Most of the machine parameters were kept constant including the printing speed of the sample’s contours 50 mm/s and infill printing speed 80 mm/s. The temperature of the printing nozzle was changed for each material, starting from 240 °C for the pure GPET, 250 °C for for the GPET/PC(75/25) and (50/50) sample, 270 °C for the GPET/PC(25/75) material. Due to high viscosity of the pure PC filament, even at 285 °C, smooth printing was impossible, therefore the pure PC samples were prepared by injection molding. Cubic samples with a side length of 50 mm were prepared to assess the accuracy of the printed model geometry.

### 2.3. Measurements and Characterization

The mechanical properties of the printed specimens were evaluated through static tensile/flexural measurements and notched Izod impact tests. The static tests were performed with the use of a Zwick/Roell Z010 universal testing machine (Zwick Roell, Ulm, Germany). The measurements were conducted according to ISO 527 standard for the tensile test, using a 1A type test specimen. The gauge length was 50 mm, while the crosshead speed was 10 mm/s. Flexural measurements were taken using standard test samples, dimensions of 4 mm × 10 mm × 80 mm according to ISO 178 standard. The span distance was 64 mm, while the test speed was 2 mm/min. Notched Izod impact tests were carried out using a Caest 9050 pendulum, where the hammer energy was 5 J. All tests were performed on a notched sample, with the notch depth of 2 mm. For comparison purposes there were two types of notches—one printed and second cut on a sample. For all mechanical measurements a minimum number of 5 samples was used for testing. 

The thermomechanical properties were measured with the use of the DMTA analysis. The tests were conducted using an Anton Paar MCR 301 rheometer (Anton Paar GmbH, Graz, Austria) attached with torsion clamps. The equipment allowed to perform the test using solid rectangular samples measuring 50 mm × 10 mm × 4 mm. The tests were conducted from the room temperature (≈30 °C) up to 170 °C, with the heating rate of 2 °C/min. The strain amplitude was 0.01% and the deformation frequency 1 Hz. The results were collected in the form of storage modulus G’ and tan δ plots.

The rheological characteristic of the prepared blends was performed with the use of a rotational rheometer Anton Paar MCR 301. Small amplitude oscillated shear measurements were performed with the use of parallel plate geometry, with the gap distance of 1mm and a 25 mm of the plate diameter. The frequency sweep measurements were conducted at 260 °C. The constant strain amplitude was set to 0.5%, while the frequency range varied from 0.1 to 100 rad/s. In order to determine the linear viscoelastic region (LVR) an initial strain sweep measurement was performed. The results of viscoelastic properties were collected in the form of storage/loss modulus and complex viscosity plots.

The structural analysis of the composites was conducted using a scanning electron microscope-Carl Zeiss EVO 40 (Jena, Germany). The samples were obtained from the fractured impact specimens. Before analysis the observed surface was coated with a thin layer of gold. 

An optical scanner Atos Core (GOM, Braunschweig, Germany) integrated with the measuring stand was used for the evaluation of the printed part geometry. Due to the semitransparent surface of the printed parts, before conducting the measurements the cubic samples were covered with thin matte layer of talc.

## 3. Results and Discussion

### 3.1. Mechanical Performance—Tensile/Flexural Measurements, Notched Izod Impact Tests

The list of mechanical properties presented in Table 1 includes different types of GPET/PC materials and GPET/PC(50/50) composite with 10% talc, the table also presents the results for the PET based reference sample PET/PC(50/50) blend and its composite with 10% content of talc.

Considering all static measurements, both the stiffness and strength of GPET/PC samples increases with the growing amount of PC in the blend. In the case of impact tests, only the GPET/PC (25/75) sample shows significant (double) impact strength. Interestingly, among all blends, the highest E module and strength is observed for the PET/PC (50/50) reference samples, which may be due to the favorable proportion of PET crystalline phase, whose mechanical properties usually exceed those obtained for GPET. For composite samples the highest stiffness was noticed for the GPET/PC (50/50) samples with the addition of 10% talc, while this material achieves the highest E module. Unfortunately, the tensile strength value significantly decreases, which is obviously an unfavorable feature. For composite samples based on the PET/PC (50/50) blend, most of the mechanical features remain at a level similar to unfilled samples, which suggests a slightly lower reinforcing factor than for GPET. However, this type of behavior can be treated as an advantage, while provides a more optimal balance of mechanical properties. 

It is worth noting that the mechanical properties of the obtained samples obtained depend not only on the characteristics of the polymer blends themselves but also on the adhesive phenomena at the boundaries of the individual layers of the printed material. Even for pure GPET sample most of the properties are strongly deteriorated. The results can be directly compared with those obtained for the same type of GPET investigated in our previous work, where the injection molding technique was used [42]. The tensile modulus/strength of the molded sample is reduced from 2156/50.9 MPa to 1490/31.4 for the printed ones. The reduction in the elongation at break value was most significant, from 350% to 3%. Therefore, already for pure materials it is clear that for the 3D printed samples the mechanical properties will not reflect the structural changes of the modified blends.

Due to the lack of extensive literature reports on GPET/PC blends, the results of the presented tests can be referred to PET/PC materials [9,43,44]. The studies confirm the partial miscibility of this type of blends in the molten state, therefore, despite the two-phase structure, this type of polymer system can be considered as compatible. This is also confirmed by the results of mechanical tests, where mechanical properties changes follow the rule of mixture, while for most of the polymer blends the rapid deterioration of mechanical parameters is observed [12,19].

### 3.2. Thermomechanical Ealuation—DMTA Measurements, HDT/VST Tests

**Figure 1:**
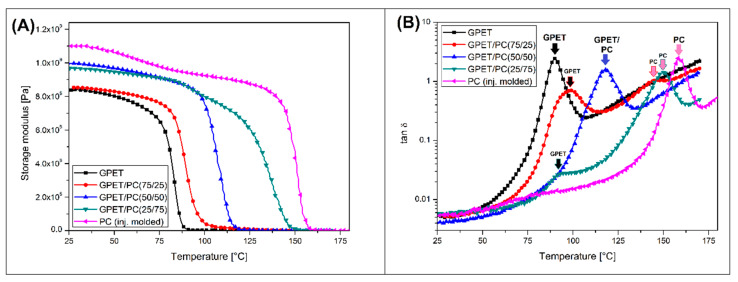
The results of the dynamic mechanical thermal analysis for polyethylene terephthalate copolymer/polycarbonate (GPET/PC) blends. (**A**) storage modulus thermograms and (**B**) tan δ curves.

The results of the DMTA analysis for GPET/PC blends shown in Figure 1 confirm the significant differences in sample stiffness caused by the addition of PC.

Both the absolute value of the storage module and its linear range are significantly improved compared to the pure GPET. The stiffness of the unmodified GPET significantly decreased already at 80 °C, while the addition of 50% PC increased this range to about 110 °C. Of course, the highest modulus values are obtained for the GPET/PC(25/75) blend but also in this case, a fairly constant decrease in the stiffness of the samples from about 110 °C is recorded. The pure PC reference sample maintains stable stiffness up to 150 °C. The conducted DMTA analysis allowed to evaluate changes of phase transition temperatures in addition to assessing the thermomechanical stability. The most important glass transition phenomenon can be observed on the tan δ graphs for the tested materials. The plots analysis clearly shows that the tan δ peak value for the pure GPET is around 90 °C. Interestingly, for the content of 25% PC, the value of the peak tan δ corresponding to GPET is shifted to 95 °C, the second slight peak observed at 145 °C refers to the presence of PC in the blend. Interestingly, for the GPET/PC(50/50) sample, only single tan δ peak was observed at 120 °C. This behavior might confirm the occurrence of miscibility of the used polymers. A further increase of the PC content to 75% causes the phase separation again, this time the tan δ peak for GPET phase reaches 95 °C, while the peak for PC phase can be observed at around 150 °C. Considering all the noticeable changes on the DMTA curves, it can be said with certainty that the thermomechanical properties of GPET/PC blends are significantly improved comparing to those obtained for the pure GPET.

**Figure 2:**
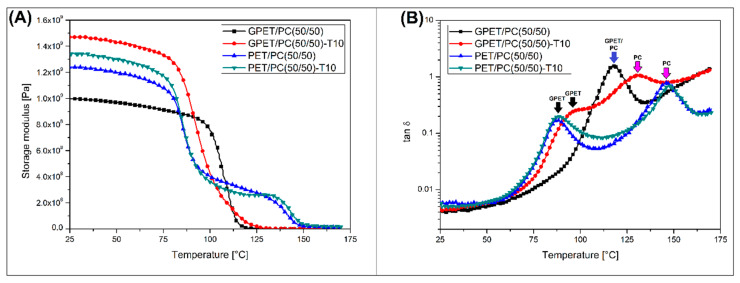
Storage modulus (**A**) and tan δ (**B**) plots for composite samples. The graphs compare the results for GPET/PC(50/50)-T10 and PET/PC(50/50)-T10 samples reinforced with 10% of talc, plots are combined with thermograms for unreinforced blends.

The DMTA analysis was also performed for the composite samples reinforced with talc particles (Figure 2).

In this case, two types of polymer blends, GPET/PC(50/50) and PET/PC(50/50) were mixed with 10% of talc (T10). The storage modulus plots presented in Figure 2A reveals a large improvement in storage modulus values for composite samples. For the GPET/PC(50/50)-T10 sample, where the stiffness was the highest, the result was unexpected because for the unreinforced GPET/PC blend the storage modulus values were significantly lower than those presented by the PET/PC blend. The direct sample comparison of the PET/PC(50/50) sample reinforced with talk allows to detect only small changes in the initial storage modulus value, while the decrease in stiffness in the glass transition range of PET and PC phase is basically similar for both materials. The changes in the characteristics of phase transitions are especially visible on the tan δ charts, where the plots for PC/PET-based materials are almost identical. The same tan δ chart for GPET/PC-based samples reveals a large difference between unfilled and talc reinforced materials. Unlike the unmodified GPET/PC blend, the GPET/PC(50/50)-T10 composite does not show full miscibility, as indicated by a single tan δ peak. Instead, there are two separate peaks, which correspond to the glass transition temperature of the GPET and PC phase. The results therefore indicate that the introduction of the fine dispersed filler like talc limits the miscibility of GPET and PC. At the same time, it is worth noting that the significant tan δ peak shift for the GPET/PC(50/50)-T10 sample still indicates the phenomenon of blend self-compatibilization, which is typically considered as a partial miscibility phenomenon. Similar behavior was observed in the case of other types of blends [45,46], where the addition of talc or chalk caused a certain reduction of miscibility for PLA-based materials. This phenomenon was described in detail by Hao et al. [47]. The addition of nanosilica caused a miscibility deterioration in the polylactic acid/poly(methyl methacrylate) PLA/PMMA system. The authors point out that the reason for phase separation was the adsorption of the PLA chain on the surface of the nanofiller. Similarly, to nanofillers, this phenomenon probably occurred in the case of the tested system with the addition of talc micro-particles. 

The results of the HDT and VST measurements support the DMTA analysis. They confirm a clear increase in thermomechanical resistance caused by the addition of PC in the blend structure (Table 2). 

In comparison to the pure GPET resin, the increase observed for the GPET/PC(50/50) sample reaches over 20 °C, while for the GPET/PC(25/75) material the HDT range increases to 116.5 °C, which is very close to the HDT of the pure PC (≈120 °C). Apparently, the addition of talc as a polymer filler does not significantly improve the HDT values, which shows that there is no need for it when thermomechanical characteristics are the key application factor. Due to the test methodology, the VST results are shifted to higher temperature values, comparing to the HDT test. However, the trends of value changes for VST are very similar to those presented by the HDT factor. It is worth noting that for the reference samples, which are made with the addition of PET instead of GPET, the values of both HDT and VST temperatures are significantly higher than for analogous materials based on the GPET/PC blend. The reason for this change is the formation of a PET crystalline phase whose thermal stability is significantly higher in comparison to the amorphous GPET. Similarly to the GPET/PC-based composite, also for the PET/PC(50/50)-T10 samples the increase in HDT and VST reaches only 3 °C in comparison to the pure blend. Therefore, in this case the addition of talc has little efficiency as well. Unfortunately, in the case of amorphous polymers, the addition of mineral fillers would have to be much larger than the used 10% [44,48]. As other research shows, it is more effective to use mineral fillers (talc, chalk, basalt etc.) for crystalline polymers [49,50,51,52].

### 3.3. Rheological Analysis—Rotational Rheometer Measurements 

**Figure 3:**
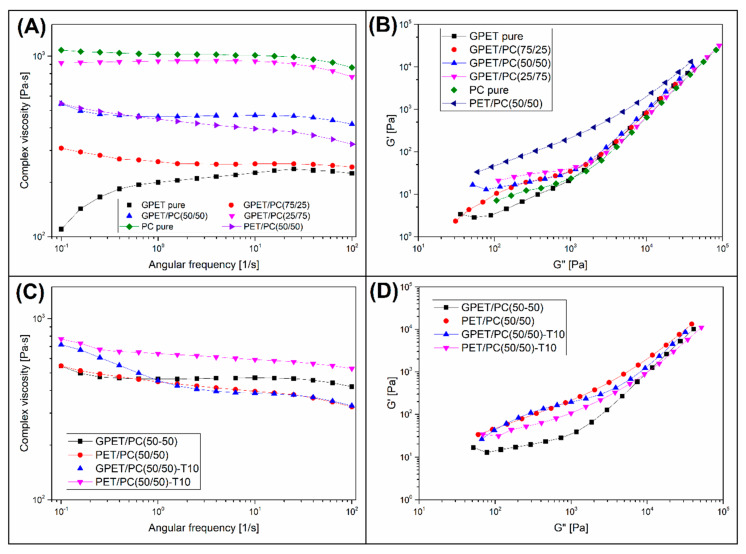
Complex viscosity and Han plots for the prepared blends (**A**,**B**) and talc reinforced composites (**C**,**D**).

Rheological measurements enabled the measurement of several important viscoelastic parameters. In addition to the complex viscosity η* diagrams in Figure 3A,D, the data from G” and G’ measurements allowed to prepare the Han plots for the obtained blends. 

The blends viscosity changes observed in the Figure 3A suggest a positive effect of the GPET addition. Even for PC rich blends containing 75% of PC the viscosity reduction is visible, while for the GPET/PC(50/50) samples the rheological characteristic is strongly modified. Interestingly, for PET/PC(50/50) blend the course of the viscosity plot was very close to GPET-based sample. The appearance of pure GPET plot indicates some restriction for this polymer, similarly to other types of thermoplastic polyesters the thermal stability is limited. This type of behavior has been already observed especially for PLA-based blends [53,54,55,56]. The relationship between the G” and G’ can be useful to determine the miscibility of the polymer blends. For GPET/PC blend the G”/G’ charts, otherwise known as Han plots, are presented in the Figure 3B. It is clear that for all GPET-based blends the slope of the curves is almost identical, especially at high modulus. This tendency indicates good phase compatibility or even their miscibility, which confirms the results of the DMTA analysis. The linear relationship disappears at low values of modulus, which is correlated with the degradation phenomenon occurring after few minutes of measurements. In contract to GPET based materials, the Han plot for PET/PC(50/50) sample does not correlate with the PC curve, indicating no phase compatibility for PET/PC system.

The complex viscosity changes for talc filled composites are compared in the Figure 3D, the plots for the reference unmodified blends are also presented. Due to the low content of the talc filler (10 wt.%), the viscosity increased only slightly. The use of fillers for the FDM technique is less common than for other processing methods, such as injection molding. Usually the content of composite additives ranges between 10–20% [57]. For the tested materials, it can be stated that the addition of talc does not cause any deterioration of the flow characteristics, however, it is worth mentioning here that it would be necessary to prepare test for materials with higher filler concentrations. The analysis of the Han plots for composite samples indicates a deterioration of the compatibility of the tested materials, as indicated by the lack of a linear correlation of the individual curves.

### 3.4. Structure Analysis—SEM Observations

**Figure 4:**
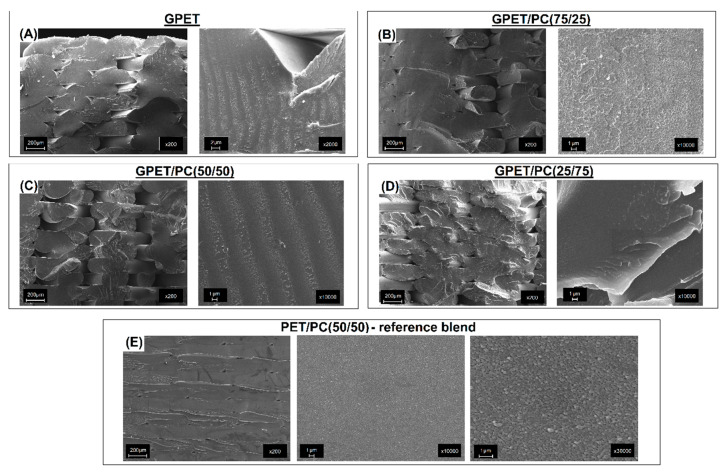
Structure analysis for different types of GPET/PC blends (**A**–**D**) and reference PET/PC sample (**E**). The observed surface was obtained from the fractured surface of the Izod sample.

The structure images presented in Figure 4 are collected from the fractured surface obtained after the impact tests. 

For all the GPET/PC blends, the obtained scanning electron microscope (SEM) images did not allow to clearly distinguish the two-phase blend structure. This fact is another confirmation of at least partial miscibility of the investigated blend system. Unlike the investigated GPET/PC blends, the reference system of PET/PC(50/50) mixture consists of two separate PET and PC phases, which are visible at high magnification. The very high fragmentation of the structure is surprising and it impedes observation even with the help of electron microscopy. The size of the dispersed phase is considerably smaller than 1 µm. Such a high fragmentation of the structure has a beneficial effect on the properties of materials; therefore, the mechanical properties of the PET/PC blend can be often better than for the GPET/PC blend.

**Figure 5:**
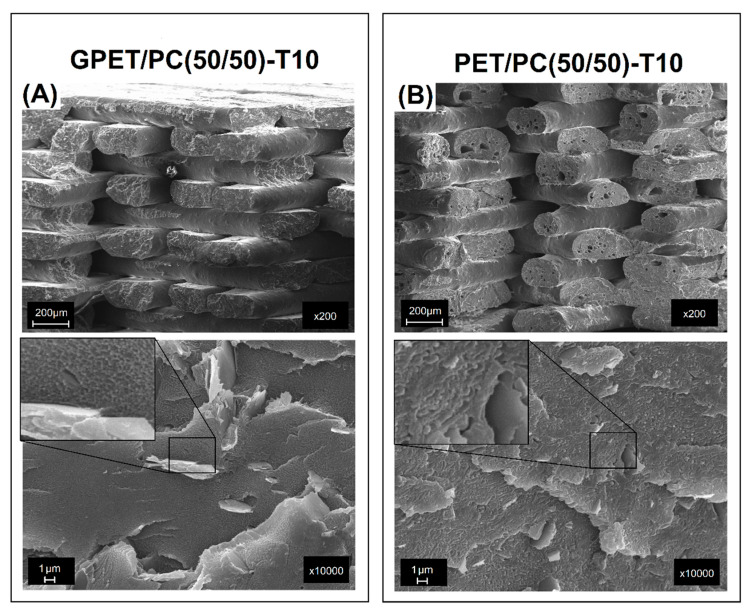
Structure analysis for the talc composite samples, where (**A**) is the GPET/PC(50/50)-T10 sample and (**B**) is the PET/PC(50/50)-T10 sample.

Two types of composites prepared with the use of GPET/PC and PET/PC blend were reinforced with 10% of the talc particles. As can be seen in Figure 5, the filler has been properly distributed in the matrix for both GPET/PC(50/50)-T10 and PET/PC(50/50)-T10 samples.

This is particularly important in the FDM technology, where the presence of agglomerates could lead to the clogging of the printing nozzle. Structure view at a high magnification for the GPET-based composite again reveals the excellent miscibility for the matrix blend, while the images obtained for PET/PC confirm phase separation. 

### 3.5. Geometry Deviations Analysis—Optical Scanning (Structured Light Method)

The quality of geometry reproduction by different materials was analyzed by comparing element frontal surface. All elements were digitized by an optical 3D scanner using structured light projection method. The application of a similar measurement method used to analyze the geometry of elements made of polymeric materials has been presented several times [27,58]. The cubic type of the sample was adopted from a study by Santana et al. [59]. The problem of warpage has been investigated several times, both in real conditions and using simulations [60,61,62]. Many research projects also describe methods to prevent this unfavorable phenomenon [63,64]. 

**Figure 6:**
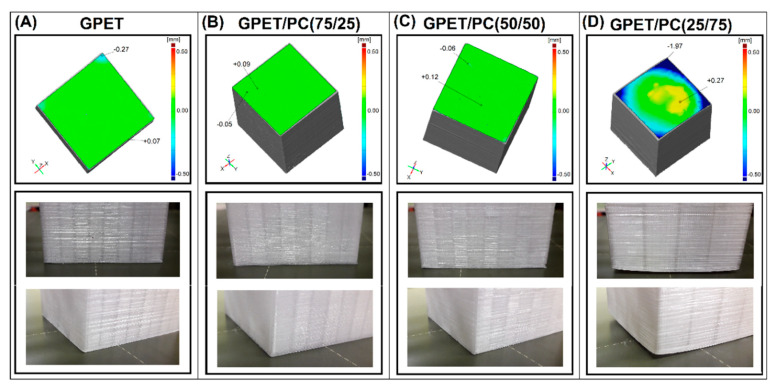
Comparison of the 3D scans and real sample deformation for different types of GPET/PC. (**A**) pure GPET sample, (**B**) GPET/PC(75/25) sample, (**C**) GPET/PC(50/50) sample and (**D**) GPET/PC(25/75) sample.

For comparison purposes the sample prepared from the pure GPET was set as reference for the other specimens. The deformation of frontal surface was considered as the critical parameter for further deliberations. On the top of the element constructed plane according to least squares method and inspected how real element surface is deformed from the one described as reference one. The pictures presented in Figure 6 show the geometry of GPET/PC blends, while the results presented in Figure 6 refer to the composite samples.

In the case of FDM-3D printing, the main type of deformation is warpage. The analysis of this defect for the tested parts was carried out by measuring the deviation of the bottom surface of the printed part in relation to the theoretical flat surface. The measurements taken with an optical scanner indicate that the flatness deviation for GPET/PC materials up to 50% PC content does not exceed 0.2–0.3 mm. This result can be considered as positive, since it shows that the deformation of the lower base of the model is almost equal to the thickness of the first layer of the printed model (≈0.2 mm). The geometric accuracy is significantly deteriorated for the PET/PC(25/75) blend, where the appearance of the samples clearly indicates an increase in the shrinkage value for subsequent layers of the model. The outer surface of the model bottom surface detaches from the work platform, which consequently causes a deformation of approximately 2 mm. An attempt to produce printed samples from the pure PC turned out to be impossible because the warpage of the printed models caused the samples to detach from the printer table. 

**Figure 7:**
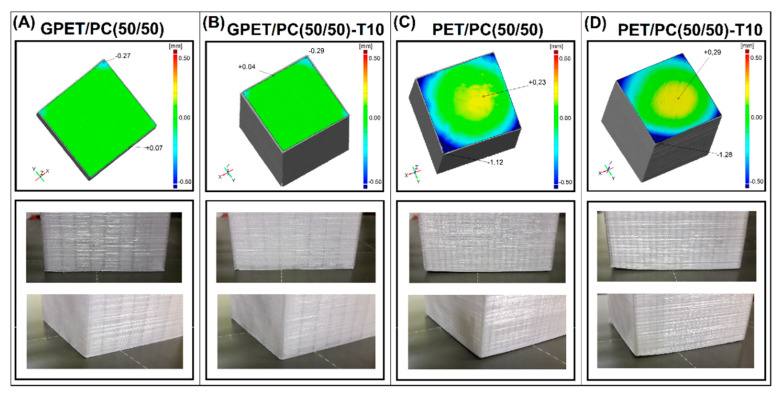
Comparison of the 3D scans and real sample deformation for 50/50% blends and composites. (**A**,**B**) GPET based materials and (**C**,**D**) PET based samples.

The geometric analysis for the composite samples includes the measurement comparison for the GPET/PC and PET/PC based samples (see Figure 7).

The results for talc-reinforced samples are compared with the unmodified blends. The addition of talc particles to the GPET based blend did not cause any visible differences in the quality of the printed part. What is worth emphasizing, for such a low filler content (10%), the transparency of the material has been limited, also the surface has become less glossy. It can be concluded that, apart from significant changes in the mechanical properties, the addition of talc does not cause negative changes in the quality of parts printed from the GPET/PC blends.

In the case of the reference samples prepared from PET/PC blends, the geometry accuracy is significantly worse. The addition of PET, as well as excessive PC content in the mixture, increases the shrinkage of the material, which leads to large warping. For PET modified blends, the reason for the excessive increase in shrinkage is the crystallization of the material structure. The crystallization phenomenon does not occur for GPET, which is the reason for the fundamental differences in the properties of these two materials. Interestingly, in the case of PET/PC(50/50)-T10 composites, model deformation is very similar to that obtained for the unreinforced material, which means that the addition of 10% talc did not cause significant changes in the crystallization mechanism.

## 4. Conclusions

The mechanical properties tests that we conducted confirm that as the percentage of PC in the blends increases, its parameters, that is, tensile/flexural strength and modulus also increase. Considering the balance of the mechanical, thermal and rheological properties of the prepared materials, the GPET/PC(50/50) blends were characterized by the best properties. The thermal resistance of the PET/PC(50/50) samples was even higher, however, given the intended use as 3D printing materials, the addition of the PET phase significantly worsens the tendencies to shrinkage and deformation of printed models. The addition of talc particles strongly increases the stiffness of the GPET based blend, while the properties of the PET/PC(50/50)-T10 composites changed only slightly. 

The miscibility of GPET/PC systems, which in practice means that there is no need to use compatibilizers or reactive processing for blends of this type, is a noteworthy phenomenon. The properties of miscible blends are usually directly proportional to the content of individual polymers, according to the rule of mixture.

In summary, it is worth emphasizing that the main goal of the conducted research work study was to facilitate the “home production” of strong, heat resistant and precise prototypes, which will allow the 3D printing technique to become even more widespread. In the future, the GPET/PC blends may become a commonly used material for the FDM printing method.

## Figures and Tables

**Table 1:** The results of mechanical test for static tensile and flexural method and notched Izod impact resistance test.

Sample	Tensile Test			Flexural Test		Izod Test
	Modulus [MPa]	Strength [MPa]	Elongation at Break [%]	Modulus [MPa]	Strength [MPa]	Impact Strength kJ/m^2^
Unmodified blends						
GPET	1490 ± 49	31.4 ± 2.1	3.0 ± 0.3	1720 ± 128	53.4 ± 5.2	1.0 ± 0.2 (1.7 ± 0.9) *
GPET/PC(75/25)	1610 ± 29	28.8 ± 5.0	2.2 ± 0.6	1730 ± 49	52.5 ± 1.2	2.8 ± 0.9 (1.1 ± 0.2)
GPET/PC(50/50)	1900 ± 177	40.3 ± 3.5	2.8 ± 0.2	2150 ± 28	64.0 ± 2.8	2.5 ± 0.5 (1.8 ± 0.5)
GPET/PC(25/75)	1990 ± 159	41.2 ± 7.3	2.7 ± 0.2	2230 ± 230	67.1 ± 4.7	4.5 ± 0.8 (4.2 ± 0.1)
PC-injection molded	2385 ± 41	58.5 ± 0.6	37.8 ± 8.0	2350 ± 57	72.3 ± 1.7	35.0 ± 4.7
PET/PC(50/50)-ref.	2320 ± 103	51.3 ± 2.9	2.7 ± 0.3	2460 ± 85	73.0 ± 2.7	4.1 ± 0.3 (1.8 ± 0.5)
Talc composites (10%)						
GPET/PC(50/50)-T10	2660 ± 183	27.0 ± 3.7	1.1 ± 0.1	3090 ± 43	34.1 ± 0.8	1.4 ± 1.1 (1.0 ± 0.5)
PET/PC(50/50)-T10	2340 ± 71	40.6 ± 1.2	2.7 ± 0.3	2390 ± 74	63.8 ± 2.7	2.3 ± 0.1 (1.5 ± 0.3)

* the impact strength values from the brackets are obtained from the samples prepared by mechanical notching.

**Table 2:** The results of head deflection temperature (HDT) and Vicat softening temperature (VST) for all types of prepared samples.

Sample	HDT (1.8 MPa) [°C]	VST (10 N) [°C]
Unmodified blends		
GPET	73.3 ± 0.4	79.6 ± 0.9
GPET/PC(75/25)	75.0 ± 1.3	99.3 ± 0.6
GPET/PC(50/50)	94.5 ± 0.7	109.0 ± 0.8
GPET/PC(25/75)	116.5 ± 0.5	136.9 ± 0.4
PC-injection molded	120.3 ± 0.9	148.3 ± 0.6
PET/PC(50/50)-ref.	101.6 ± 4.2	140.5 ± 0.6
Talc composites (10%)		
GPET/PC(50/50)-T10	88.6 ± 0.3	109.9 ± 0.4
PET/PC(50/50)-T10	104.5 ± 1.8	143.6 ± 0.6
